# PPARs as Metabolic Regulators in the Liver: Lessons from Liver-Specific PPAR-Null Mice

**DOI:** 10.3390/ijms21062061

**Published:** 2020-03-17

**Authors:** Yaping Wang, Takero Nakajima, Frank J. Gonzalez, Naoki Tanaka

**Affiliations:** 1 Department of Metabolic Regulation, Shinshu University School of Medicine, Matsumoto, Nagano 390-8621, Japan; 16mh281b@shinshu-u.ac.jp (Y.W.); nakat@shinshu-u.ac.jp (T.N.); 2 Laboratory of Metabolism, National Cancer Institute, National Institutes of Health, Bethesda, MD 20892, USA; gonzalef@mail.nih.gov; 3 Research Center for Social Systems, Shinshu University, Matsumoto, Nagano 390-8621, Japan

**Keywords:** PPAR, NAFLD, NASH, insulin resistance, liver fibrosis

## Abstract

Peroxisome proliferator-activated receptor (PPAR) α, β/δ, and γ modulate lipid homeostasis. PPARα regulates lipid metabolism in the liver, the organ that largely controls whole-body nutrient/energy homeostasis, and its abnormalities may lead to hepatic steatosis, steatohepatitis, steatofibrosis, and liver cancer. PPARβ/δ promotes fatty acid β-oxidation largely in extrahepatic organs, and PPARγ stores triacylglycerol in adipocytes. Investigations using liver-specific PPAR-disrupted mice have revealed major but distinct contributions of the three PPARs in the liver. This review summarizes the findings of liver-specific PPAR-null mice and discusses the role of PPARs in the liver.

## 1. Introduction

Administration of Wy-14643, nafenopin, and fibrate drugs to rodents results in hepatic peroxisome proliferation. These agents are thus designated as peroxisome proliferators (PPs) [1]. To explain a mechanism of rapid and drastic changes following PP administration, the involvement of transcription factors was assumed and the first peroxisome proliferator-activated receptor (PPAR, later defined as PPARα (NR1C1)) was identified in 1990 [2]. Subsequently, two other PPARs, PPARβ/δ (NR1C2) and PPARγ (NR1C3), were identified [3,4]. 

PPARs are activated by various ligands. For example, long-chain fatty acids (FAs) and eicosanoids are typical endogenous ligands for PPARα and PPARβ/δ [5,6], whereas PPARγ is activated by arachidonic acid metabolites, such as 5-oxo-15(S)-hydroxyeicosatetraenoic acid and 5-oxo-eicosatetraenoic acid [7,8]. Many exogenous compounds (e.g., lipid-lowering fibrates) and several endocrine-disrupting chemicals, such as pesticides (diclofap-methyl, pyrethins, and imazalil), herbicides (2,4-dichlorophenoxyacetic acid), and phthalate esters (diethylhexyl phthalate) possess PPARα-activating properties in rodents [9,10]. Wy-14643 is a potent PPARα activator that is widely used in experimental studies, but has some modest low-affinity PPARβ/δ- and PPARγ-activating potential [4]. GW501516 is a highly-selective PPARβ/δ ligand, and anti-diabetic thiazolidinedione (TZD) derivatives, such as pioglitazone, GW1929, and GW2090, are specific PPARγ activators [11]. 

The expression of the three PPARs is quite different between organs, indicating their distinct physiological roles. Notably, PPARα is expressed in hepatocytes, cardiomyocytes, proximal renal tubular cells, and brown adipocytes. PPARβ/δ is more ubiquitous but mainly found in skeletal muscle, skin, adipose tissue, heart, liver, and inflammatory cells. PPARγ has three splicing variant isoforms (γ1, γ2, and γ3) that display differences in tissue localization for each isoform despite the same DNA binding specificity: γ1 (ubiquitous localization), γ2 (localized in adipose tissue), and γ3 (localized in macrophages, colon, and adipose tissue). The transcriptional activity of PPARγ2 is 5-10 times greater than that of PPARγ1 [12]. When PPARα is activated by FA, FA β-oxidation and ensuing adenosine triphosphate (ATP) production and ketogenesis are enhanced, thus indicating that PPARα is the main controlling factor for FA oxidation and energy generator under a nutrient-deprived state. On the contrary, when PPARγ is activated, several proteins, such as FA-binding protein 4 (FABP4, also known as aP2), are up-regulated and FAs are stored in adipocytes as triacylglycerol (TAG). Under conditions of nutrient overload and obesity, PPARγ is induced and activated in liver where it is involved in FA storage as lipid droplets [13,14]. Therefore, differences of PPARs in cell-specific expression, ligands, and target genes suggest a major but distinct contributions of the three PPARs to energy/nutrient metabolism in the liver. 

Genetically-modified mice using Cre-LoxP system are useful tools for evaluating cell-specific roles of PPARs in vivo. This review summarized the recent findings of liver-specific PPAR-null mice, updated the knowledge regarding the role of PPARs in the liver, and discussed unsolved questions. All descriptions in this review refer to mouse PPARs unless otherwise specified. 

## 2. Role of PPARα in the Liver

**Figure 1:**
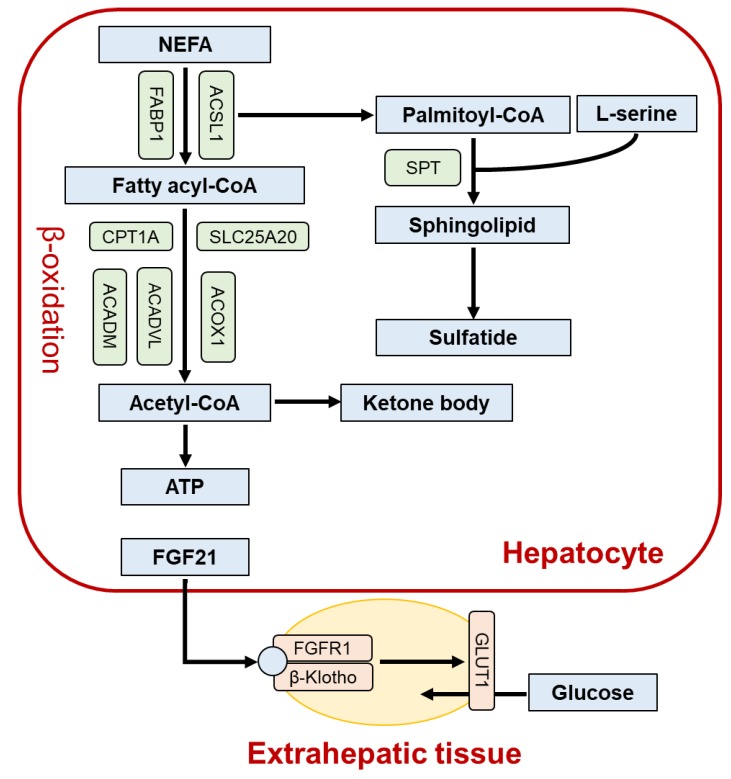
The role of peroxisome proliferator-activated receptor α (PPARα) in hepatic lipid metabolism. Enzymes/proteins up-regulated by PPARα activation are indicated as green boxes. ACADM, medium-chain acyl-coenzyme A (CoA) dehydrogenase; ACADVL, very-long chain acyl-CoA dehydrogenase; ACOX1, acyl-CoA oxidase 1; ACSL1, acyl-CoA synthetase long chain family member 1; ATP, adenosine triphosphate; CPT1A, carnitine palmitoyltransferase I; FABP1, fatty acid-binding protein 1; FGF21, fibroblast growth factor 21; FGFR1, fibroblast growth factor receptor 1; GLUT1, glucose transporter 1; NEFA, non-esterified fatty acid; SLC25A20, solute carrier family 25 member 20; SPT, serine palmitoyltransferase.

Constitutive mitochondrial β-oxidation activity was significantly reduced in the livers of mice lacking PPARα gene (*Ppara*-null mice) [15]. The fact that medium-chain acyl-coenzyme A (CoA) dehydrogenase and acyl-CoA oxidase 1, the rate-limiting enzymes in mitochondrial and peroxisomal FA β-oxidation, respectively, are PPARα target genes prompts consideration of a crucial role for PPARα in FA catabolism and clearance (Figure 1). Polyunsaturated FAs (PUFAs) are endogenous activators of PPARα [5,6], and when mice are fed a high-fat diet (HFD) without PUFAs for 5 weeks, they show high serum FA levels and severe fatty liver. Supplementation of the HFD with PUFAs, mainly linoleic acid and α-linolenic acid, ameliorated fatty liver due to PPARα activation and enhanced hepatic FA β-oxidation [16]. 

PPARα is also involved in sphingolipid metabolism in the liver. Sphingolipids are a class of the complex lipids, having a ceramide structure, and are essential for diverse biological processes including development and growth. Sphingolipids are synthesized de novo, which is initiated from a condensation of L-serine and palmitoyl-CoA through serine palmitoyltransferase (SPT). SPT is a rate-limiting enzyme in overall sphingolipid biosynthesis, and PPARα activation increases SPT gene expression in human skin cells and mouse livers [17,18]. PPARα also controls palmitoyl-CoA synthesis by activating the gene encoding long-chain acyl-CoA synthetase [15]. Thus, PPARα participates in regulation of the first step in sphingolipid biosynthesis by modulating SPT enzyme and palmitoyl-CoA levels. 

PPARα controls the synthesis of a certain sphingolipid class, sulfated glycolipid (sulfatide). Fenofibrate administration to mice up-regulated the expression of the gene encoding the sulfatide-generating enzyme cerebroside sulfotransferase in a PPARα-dependent manner [19], resulting in increases in liver and serum sulfatide contents [20]. Because sulfatide has an anti-thrombotic effect [21], PPARα activators might be useful for preventing cardiovascular diseases through induction of serum sulfatide production. 

PPARα also reflated the gene encoding fibroblast growth factor 21 (FGF21), a hepatokine secreted from the liver into blood, binding to a plasma membrane receptor complex on target tissues, mainly the FGF receptor 1 and β-Klotho heterodimer, and enhancing expression of glucose transporter 1 in extrahepatic tissues, leading to improved systemic insulin sensitivity and lipid turnover [22,23]. Hepatic and serum levels of FGF21 were significantly increased by PPARα activation, whereas the constitutive levels were markedly lower in *Ppara*-null mice compared with wild-type mice [24]. These findings suggest that PPARα’s control of hepatic metabolism is partially mediated by FGF21. 

In response to prolonged fasting, circulating ketone bodies and FGF21 are increased. Recently, ketone bodies have attracted attention as not only an energy source of the brain, but also a substance correcting metabolic disease and preventing aging and cancer [25]. Expression of hydroxymethylglutaryl-CoA synthase 2, a rate-limiting enzyme of ketogenesis, is increased by PPARα activation, and adaptive ketogenesis is impaired in *Ppara*-null mice [26]. 

Hepatic steatosis without ethanol consumption is designated as non-alcoholic fatty liver disease (NAFLD), which includes severe disease phenotype non-alcoholic steatohepatitis (NASH). NAFLD/NASH may progress to liver cirrhosis and hepatocellular carcinoma, and PPARα may influence the severity of NAFLD/NASH [27,28,29,30,31,32,33,34]. In previous studies using liver tissues of NAFLD/NASH patients, PPARα expression was lower with progression of fibrosis [34,35]. Hepatic PPARα and circulating ketone bodies, indicators of FA utilization, were significantly low in hepatic steatosis/steatohepatitis in patients with citrin deficiency, a relatively common urea cycle disorder in the Asian population [36,37]. PPARα also has anti-inflammatory properties through counteracting nuclear factor kappa B (NF-κB) and enhancing FGF21 [22,23,38]. Previous studies suggest that PPARα exerts anti-tumor effects in hepatoma cells by regulating NF-κB signaling [39]. Furthermore, oleoylethanolamide, an endocannabinoid-like endogenous molecule that binds PPARα with high affinity, ameliorated thioacetamide-induced hepatic fibrosis in a PPARα-dependent manner [40]. These findings indicate that PPARα ligands may exhibit not only anti-steatotic effects, but also anti-inflammatory and anti-fibrotic effects.

## 3. Lessons from Liver-Specific *Ppara*-Null Mice

Genetically modified mice using Cre-LoxP system provide useful information about cell-specific roles of PPARs in vivo. Although several PPARα activators induce hepatomegaly and cell proliferation, it was unclear which hepatic cell type was mainly responsible for the function of PPARα in lipid homeostasis and phenotypic changes following PP administration, such as hepatomegaly, hepatocyte proliferation, and liver cancer. Hepatocyte *Ppara* (*Ppara*^ΔHEP^) and macrophage *Ppara* (*Ppara*^ΔMAC^)-specific knockout mice were treated with Wy-14,643 for 14 days. Hepatomegaly and hepatocyte proliferation were observed in wild-type and *Ppara*^ΔMAC^ mice, but not *Ppara*^ΔHEP^ and whole-body *Ppara*-null mice. On the contrary, down-regulation of pro-inflammatory cytokines interleukin (IL)-15 and IL-18 following Wy-14,643 treatment were dependent on the presence of macrophage PPARα. These results indicate the importance of cell specificity of PPARα in its function [41]. 

Under the circumstance in which FAs are predominantly used as an energy source, such as fasting, *Ppara*-null mice cannot augment hepatic FA catabolism in response to increased FA influx from adipose tissue, resulting in severe hepatosteatosis [26]. *Ppara*^ΔHEP^ mice also exhibited more severe hepatic steatosis during fasting; ageing; and two NAFLD/NASH models, methionine- and choline-deficient diet and HFD feeding. Hepatic PPARα activity was influenced by circulating FA through adipocyte lipolysis. During fasting, hepatocyte PPARα disruption could not adjust liver metabolism to excess FA release from adipose tissues, resulting in aggravation of hepatic steatosis [42]. This adaptive response may be partially mediated by hepatokines, such as insulin-like growth factor-binding protein 1 and growth differentiation factor 15 [43]. 

The role of hepatocyte PPARα for hepatic steatosis and liver-adipose axis was also intensively evaluated [44]. After a 24 h fasting cycle, hepatic FA accumulation was less in *Ppara*^ΔHEP^ mice compared to *Ppara*-null mice, although hepatic PPARα target genes were similarly suppressed in both mice. In *Ppara*^ΔHEP^ mice, the expression of genes encoding FA β-oxidation enzymes and lipase activity were increased in brown adipose tissue, heart, and muscle. Therefore, when hepatic PPARα is disrupted, extrahepatic PPARα activity compensates to increase and utilize excess lipid, thus protecting livers against fasting-induced steatosis [44]. 

## 4. Role of PPARβ/δ in the Liver

Activation of PPARβ/δ may prevent dyslipidemia, insulin resistance, obesity, and NAFLD [45]. Compared to other two PPARs, PPARβ/δ expression levels are far higher in muscle and PPARβ/δ is activated in muscle during the fed state and with exercise, increasing fuel consumption and β-oxidation [46]. PPARβ/δ activation increases liver glucose catabolism by inducing glucose 6-phosphate dehydrogenase activity, inhibiting liver glucose output, suppressing FA release from white adipose tissue, and promoting β-oxidation in muscle. Mice lacking PPARβ/δ gene (*Ppard*-null mice) had lower metabolic activity and glucose intolerance uniformly [47]. 

Metabolic syndrome-related dyslipidemia is mainly caused by liver overproduction of TAG-containing lipoproteins. PPARβ/δ agonists showed a strong TAG-decreasing effect, which amplifies the PPARγ coactivator 1α pathway by the restoration of adenosine monophosphate-activated protein kinase (AMPK) activity in the liver [48]. In obese monkey, PPARβ/δ activation by the potent PPARβ/δ agonist GW501516 normalized serum insulin and TAG concentrations, decreased low-density lipoprotein cholesterol, and increased high-density lipoprotein cholesterol [49], which was also the case in diet-induced and genetically obese mice [50]. Although PPARβ/δ mediates the transcriptional response to native very low density lipoprotein (VLDL) in macrophage [51], *Ppard*-null mice were found to exhibit up-regulation of hepatic VLDL receptor due to activation of heme-regulated eukaryotic translation initiation factor 2α kinase and nuclear factor erythroid 2-related factor 2 [52]. 

**Figure 2:**
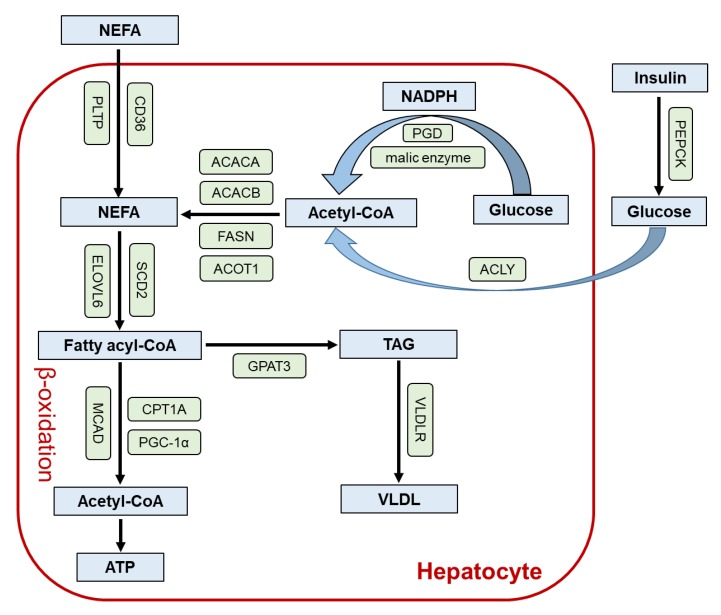
The role of PPARβ/δ in hepatic lipid metabolism. Enzymes/proteins up-regulated by PPARβ/δ activation are indicated as green boxes. ACACA, acetyl-CoA carboxylase 1; ACACB, acetyl-CoA carboxylase 2; ACLY, ATP citrate lyase; ACOT1, acyl-CoA thioesterase 1; CD36, cluster of differentiation 36; CPT1A, carnitine palmitoyltransferase I; ELOVL6, elongation of very long chain fatty acid-like family member 6; FASN, fatty acid synthase; GPAT3, glycerol-3-phosphate acyltransferase 3; NADPH, nicotinamide adenine dinucleotide phosphate; NEFA, non-esterified fatty acid; PEPCK, phosphoenolpyruvate carboxykinase; PGC-1α, peroxisome proliferator-activated receptor gamma coactivator 1-alpha; PGD, phosphogluconate dehydrogenase; PLTP, phospholipid transfer protein; SCD2, stearoyl-CoA desaturase 2; TAG, triacylglycerol; VLDL, very low density lipoprotein; VLDLR, VLDL receptor.

The contribution of PPARβ/δ to hepatic lipid metabolism is summarized in Figure 2, but is somewhat controversial [53]. Intravenous injection of adenovirus with PPARβ/δ complementary DNA (cDNA) into *db/db* mice resulted in activation of sterol regulatory element-binding protein (SREBP)-1, up-regulation of lipase, and improved liver steatosis [54]. In primary mouse hepatocytes and livers from HFD-fed mice, the PPARβ/δ agonist GW1516 up-regulated PPARβ/δ-specific target genes *Adfp* and *Cpt1a* and enhanced FA oxidation. GW1516 also inhibited hepatic lipogenesis through activating AMPK and suppressing SREBP-1c [55]. Nevertheless, the other potent PPARβ/δ agonist GW501516 increased the expression of lipogenic enzyme acetyl-CoA carboxylase (ACC) 2 and resultantly augmented liver TAG content in *db/db* mice, as done in reference [47]. It remains unclear whether these differences between GW1516 and GW501516 are derived from ligand affinities, specificities toward PPARβ/δ, metabolism/distribution of the compounds, or other factors. 

Similar to PPARα, PPARβ/δ possesses anti-inflammatory effects in the liver by inhibiting NF-κB activity by directly binding to its subunit p65 [56,57,58]. In hepatitis B virus transgenic mice, PPARβ/δ activation in Kupffer cells ameliorated inflammation and prevented steatosis-induced hepatic tumorigenesis [59]. PPARβ/δ agonist L165041 and GW501516 increased hepatic expression of fibrosis markers in carbon tetrachloride (CCl_4_)-injected mice [60,61], but not the agonist KD3010. Therefore, PPARβ/δ-specific agonists are attractive because PPARβ/δ activation can attenuate dyslipidemia, insulin resistance, hepatic steatosis, and inflammation [47,48,62,63,64]. 

Although preclinical data on PPARβ/δ agonists in metabolic diseases are quite encouraging, some conflicting but serious observations were found, bringing its utility as a drug target into question. GW501516-treated mice developed adenocarcinoma [65] and the PPARβ/δ agonist L-165041 increased hepatotoxicity due to hepatic stellate cell (HSC) activation [60]. However, the role of PPARβ/δ in carcinogenesis is controversial because conflicting studies indicate that PPARβ/δ inhibits and promotes tumorigenesis. It was proposed that heterogeneity in PPARβ/δ expression in cell types during tumorigenesis and the existence of natural PPARβ/δ agonists/antagonists and inverse agonists render the analysis of PPARβ/δ in cancer complex and controversial [66]. On the other hand, PPARβ/δ antagonism and inverse agonism have begun to attract attention, such as covalent antagonists GSK3787 and CC618 that were reported in studies on psoriasis and breast cancer, but the role in the liver is still unknown [67,68]. 

A dual PPARα/β activator elafibranor (GFT505) was developed, which showed beneficial effects for human NASH. In this GOLDEN-505 study, patients with NASH resolution after receiving elafibranor 120 mg had reduced liver fibrosis stages compared with those without NASH resolution, and liver enzymes, lipids, glucose profiles, and markers of systemic inflammation were significantly reduced in the elafibranor 120 mg group compared with the placebo group. Elafibranor was well tolerated without causing cardiac events or weight gain, but did produce a mild reversible increase in serum creatinine [69]. PPARα/β dual regulators will be a major topic of basic scientific and clinical research in the coming years, thereby considering PPARα/β activators for treatment of NASH in humans.

## 5. Lessons from Liver-Specific *Ppard*-Null Mice

Liu et al. examined the role of hepatic PPARβ/δ using an adenovirus-mediated PPARβ/δ overexpression and hepatocyte-specific *Ppard*-null (*Ppard*^ΔHEP^) mice [70]. Hepatic PPARβ/δ overexpression increased the expression of ACC1 but increased FA uptake and β-oxidation in isolated soleus muscle, leading to decreased circulating TAG and non-esterified FA levels. On the contrary, liver-specific ACC1 knockdown reduced hepatic TAG content but decreased FA uptake in muscles, suggesting linkage between hepatic PPARβ/δ, hepatic lipogenesis, and muscle FA utilization. In *Ppard*^ΔHEP^ mice, hepatic induction of ACC1, ACC2, fatty acid synthase, and stearoyl-CoA desaturase 1 and increased muscle FA uptake during the dark cycle was abolished, suggesting that PPARδ regulates circadian changes in hepatic lipogenic activity and muscle FA uptake. Indeed, increased expression of cluster of differentiation (CD) 36 and FABP3 in the muscle by PPARβ/δ ligand GW501516 was completely diminished in *Ppard*^ΔHEP^ mice. 

To further investigate the interconnection between hepatic PPARβ/δ, hepatic lipogenesis, and muscle FA uptake, lipidomic analyses were performed. Liu et al. identified that phosphatidylcholine (PC) (18:0/18:1) (1-stearoyl-2-oleoyl-sn-glycero-3-phosphocholine) levels were increased by hepatic PPARβ/δ overexpression but decreased in *Ppard*^ΔHEP^ mice [70]. Increased PC (18:0/18:1) levels by GW501516 treatment were diminished in *Ppard*^ΔHEP^ mice, and PC (18:0/18:1) treatment to myotubes increased FA uptake and CD36 and FABP3 expression. Furthermore, the effect of PC (18:0/18:1) on promoting FA utilization in muscle was abolished in *Ppara*-null mice and *Ppara*-depleted myotubes. Collectively, *Ppard*^ΔHEP^ mice identified a novel role of hepatic PPARβ/δ that enhances hepatic synthesis, muscle FA utilization, and improved dyslipidemia through a metabolic network between hepatic PPARδ–PC (18:0/18:1)–muscle PPARα. 

## 6. Role of PPARγ in the Liver

PPARγ is highly expressed in adipose tissue and macrophages, and plays important roles in adipogenesis, lipid metabolism, insulin sensitivity, and immune regulation [71]. PPARγ is expressed at much lower levels in the liver and muscle, but shows relatively high expression in the placenta [72]. In the past two decades, PPARγ has been a focus of attention as a transcription factor associated with metabolic syndrome. 

**Figure 3:**
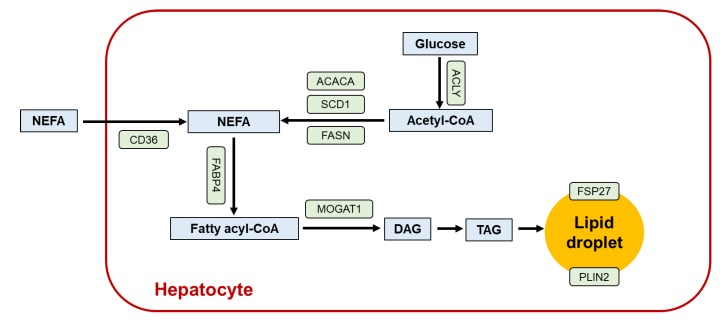
The role of PPARγ in hepatic lipid metabolism. Enzymes/proteins up-regulated by PPARγ activation are indicated as green boxes. CD36, cluster of differentiation 36; DAG, diacylglycerol; FABP4, fatty acid binding protein 4; FSP27, fat-specific protein 27 (cell death-inducing DNA fragmentation factor A-like effector c); MOGAT1, monoacylglycerol O-acyltransferase 1; NEFA, non-esterified fatty acid; PLIN2, perilipin 2; TAG, triacylglycerol.

The contribution of PPARγ to hepatic lipid metabolism is summarized in Figure 3. When PPARγ is ectopically overexpressed in hepatocytes, lipid droplets emerge. Adenovirus-mediated overexpression of PPARγ2 in hepatocytes increased hepatosteatosis and hepatocyte-specific disruption of PPARγ gene (*Pparg*) decreased liver steatosis in *ob/ob* mice [13,73]. The notion that hepatic PPARγ expression promotes steatosis is supported by the facts that PPARγ up-regulates several proteins associated with lipid uptake, TAG storage, and formation of lipid droplets, such as FABP4, fat-specific protein 27 (FSP27)/Cidec, CD36, monacylglycerol O-acyltransferase 1, and perilipin 2 [74]. When the expression of adipogenic transcriptional factor genes *Pparg* and *Srebp1c* were observed during HFD feeding, *Pparg2* mRNA levels rose before *Srebp1c* mRNA at 4 weeks [75], which were closely associated with adipogenesis-related protein, CD36, and fatty acid synthase [76,77]. A significant increase in hepatic PPARγ is one of the common phenotypes of steatotic animals associated with obesity [13] and without obesity, which lack TAG-storing capacity in adipocytes [78,79]. Similar overexpression of hepatic PPARγ was observed in NAFLD/NASH patients after pancreaticoduodenectomy, who were generally non-obese [80,81].

Although activation of PPARγ is steatogenic, treating genetically obese or diet-induced NAFLD/NASH mice with PPARγ ligands decreases hepatic TAG. This distinct effect mainly stems from enhanced synthesis of adiponectin in adipose tissue [82]. Circulating adiponectin could increase glucose uptake and FA oxidation in hepatocytes by activating AMPK, thereby improving systemic insulin sensitivity and reducing liver steatosis [83]. PPARγ has significant anti-inflammatory properties, which can regulate the immune inflammatory response [84]. PPARγ activation reduced inflammatory response by negatively interfering with NF-κB and signal transducers and transcriptional activators [85], suppressed production of tumor-necrosis factor α and IL-1β in monocytes and macrophages, and polarized into anti-inflammatory M2 macrophage [86]. Compared with hepatocytes and Kupffer cells, PPARγ2 is highly expressed in quiescent HSC, and PPARγ is down-regulated during HSC activation [87]. PPARγ agonists could reduce hepatic fibrosis by restraining HSC proliferation and driving activated HSC to apoptosis [88]. 

## 7. Lessons from Liver-specific *Pparg*-null Mice

In *Pparg*^ΔHEP^ mice, expression of genes associated with adipogenesis and FA uptake were down-regulated. Paradoxically, exacerbation of systemic insulin resistance, adiposity, and hyperlipidemia against reduced hepatic steatosis in diabetic *Pparg*^ΔHEP^ mice corroborated that liver PPARγ protects other tissues from TAG accumulation and insulin resistance [89]. Lipoatrophic A-ZIP/F-1 mice disrupting hepatocyte *Pparg* reduced hepatic steatosis but aggravated hyperlipidemia and muscle insulin resistance [89]. Insulin resistance found in these mice was resistant to rosiglitazone, suggesting that in the absence of adipose tissue, the liver is a primary target for TZD action. In contrast, the finding that rosiglitazone remained effective in wild-type *Pparg*^ΔHEP^ mice demonstrated that adipose is the major site of TZD action in mice with normal adipose tissue. 

Rosiglitazone could inhibit NF-κB activation and hepatic fibrosis after bile duct ligation in wild-type mice, but these changes disappeared in *Pparg*^ΔHEP^ mice, suggesting a key role for PPARγ in the context of biliary fibrosis [90]. 

Macrophages accumulate in adipose tissue during obesity, promote inflammation, and impair insulin sensitivity. However, HFD-induced hepatic steatosis was milder in *Pparg*^ΔHEP^ mice compared with *Pparg*^ΔMAC^ mice [91]. Compared with *Pparg*^ΔHEP^ mice and control mice, *Pparg*^ΔMAC^ mice and mice where *Pparg* is specifically disrupted in HSC showed aggravation of hepatic necroinflammation and fibrosis after repeated injections of CCl_4_. These results revealed that PPARγ in non-parenchymal cells mainly contributes to inflammatory response and ensuing fibrogenesis in the liver [92]. 

## 8. The Other Factors Modulating Hepatic PPAR Function

Environmental factors, such as nutrients, could modify the liver PPAR activity. For example, extra-virgin olive oil with high phenolic content increased hepatic PPARα gene expression in HLA-B27 transgenic rats, exerting cholesterol-lowering effects [93]. Moreover, polyphenols from different sources (coffee, olives, rice, berries, etc.) could reduce fat mass and serum/liver lipids in HFD-fed animals [94]. Especially in liver and adipose tissue, plant-derived polyphenols have been found to increase the expression of PPARα. Catechins and linalool are key bioactive compounds found in tea. Epigallocatechin gallate was found to not only activate PPARα, but also inhibit PPARγ. Linalool also activated PPARα, enhanced FA uptake and utilization in the liver, and reduced plasma TAG [95]. Moreover, vitamin D down-regulated perilipin 2 and PPARγ and prevented lipid accumulation in muscles [96]. 

Another possible important modulators of PPAR activity is cellular stress, such as oxidative stress and endoplasmic reticulum (ER) stress. In many tissues, reactive oxygen species (ROS) activate the Wnt/β-catenin pathway, leading to PPARγ inactivation [97,98], whereas PPARγ agonists reduce ROS production and chronic inflammation and suppress the Wnt/β-catenin pathway [99]. PPARα signaling was found to prevent the induction of mitochondrial and ER stress and reduce the synthesis of apolipoprotein B, thus improving the hyperlipidemia and fatty degeneration of the liver that is related to fructose intake [100]. The association between c-jun N-terminal kinase activation and PPARα down-regulation has also been documented [37,101]. 

## 9. Conclusions—Issues to Be Solved for Contributing to Human Health

This review summarized multiple but distinct roles of PPAR in the liver, evidenced by previous experimentations using specific agonists and liver-specific PPAR-disrupted mice. There is no doubt that PPARs are intriguing therapeutic targets for metabolic syndrome, insulin resistance, dyslipidemia, and NAFLD/NASH. However, the effectiveness is still unsatisfactory and further research, improvement, and trials are needed to apply PPAR-targeted agents to human metabolic diseases. The reasons for insufficient effectiveness of PPAR activators may be derived from different roles of the three PPARs between cell types, tissues, and underlying conditions (e.g., abnormalities mainly of glucose/insulin metabolism vs. cholesterol/bile acid metabolism); different effects of PPAR modulation on systemic/hepatic metabolism; and different potencies to stimulate PPARs. Dual/triple agonists, combination of agonists/antagonists, and tissue-specific agonists/antagonists would be critical to obtain the benefits of PPAR activation while minimizing adverse metabolic effects. Furthermore, modulation of available agents to improve cell/tissue specificity and tissue delivery efficiency, as well as pathogenesis-based personalization/optimization of pharmacological interventions, might be useful to improve human health through PPARs. 

## Abbreviations

ACC	acetyl-coenzyme A carboxylase
AMPK	adenosine monophosphate-activated protein kinase
ATP	adenosine triphosphate
CCl_4_	carbon tetrachloride
CD	cluster of differentiation
cDNA	complementary DNA
CoA	coenzyme A
ER	endoplasmic reticulum
FA	fatty acid
FABP	fatty acid-binding protein
FGF21	fibroblast growth factor 21
FSP27	fat-specific protein 27
HFD	high fat diet
HSC	hepatic stellate cell
IL	interleukin
NAFLD	non-alcoholic fatty liver disease
NASH	non-alcoholic steatohepatitis
NF-κB	nuclear factor kappa B
PC	phosphatidylcholine
PP	peroxisome proliferator
PPAR	peroxisome proliferator-activated receptor
PUFA	polyunsaturated fatty acid
ROS	reactive oxygen species
SPT	serine palmitoyltransferase
SREBP	sterol regulatory element binding protein
TAG	triacylglycerol
TZD	thiazolidinedione
VLDL	very-low-density lipoprotein
